# Association of a four‐gene model with allergic diseases: Two‐year follow‐up of a birth cohort study

**DOI:** 10.1002/iid3.385

**Published:** 2020-12-05

**Authors:** Li Hua, Fen Yang, Qian Chen, Quanhua Liu, Ruoxu Ji, Haipei Liu, Jianmin Ye, Jun Zhang, Jianhua Zhang, Yixiao Bao

**Affiliations:** ^1^ Department of Pediatric Pulmonology, Xin Hua Hospital Shanghai Jiao Tong University School of Medicine Shanghai China; ^2^ Ministry of Education‐Shanghai Key Laboratory of Children's Environmental Health, Xin Hua Hospital Shanghai Jiao Tong University School of Medicine Shanghai China; ^3^ Shanghai Tonxin Pediatric Clinic Shanghai China

**Keywords:** allergic disease, birth cohort, eczema, gene

## Abstract

**Background:**

Our previous study has developed a four‐gene model involving *IL13* rs20541, *IL4* rs2243250, *ADRB2* rs1042713, and *FCER1B* rs569108 associated with asthma and atopy in Chinese Han children. However, whether the gene model is associated with allergies in early life has yet to be determined. This study aimed to apply the gene model in a birth cohort to investigate its associations with the development of allergic diseases in Chinese Han toddlers.

**Methods:**

Five hundred and ninety‐seven children from a birth cohort completing 2‐year follow‐up were included. Epidemiologic information and cord blood were collected. Children were genotyped for the above polymorphisms and divided into high or low genetic risk groups based on the genotypes. Subjects were followed at 6, 12, and 24 months, with information on allergic diseases collected via standard questionnaires and assessed by specialists.

**Results:**

Two hundred and eighty‐four children were divided into a high‐risk group and 313 into a low‐risk group. Between the two groups, a significant difference was only found in delivery mode among all the subject characteristics (*p* = .025). After stratification for delivery mode, children at high risk were more likely to develop eczema (relative risk [RR] = 1.46, *p* = .040) over 2 years of follow‐up compared with those at low risk. No significant associations were found between genetic risk and food allergy, wheezing and allergic rhinitis (*p* > .05).

**Conclusion:**

The gene model was significantly associated with the development of eczema in Chinese Han toddlers. Long‐term follow‐up along with functional and replication studies on the gene model are still needed in future.

## Graphical abstract

The four‐gene model involving IL13 rs20541, IL4 rs2243250, ADRB2 rs1042713, and FCER1B rs569108 was significantly associated with the development of eczema in Chinese Han toddlers. Long‐term follow‐up along with functional and replication studies on the gene model are still needed in future.

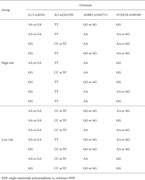

## 1 INTRODUCTION

Asthma and atopy are complex phenotypes driven by gene‐environment interactions.^1^, ^2^ Ober and Hoffjan^3^ reviewed 118 genes associated with asthma or atopy, and found that 10 of them had been replicated in >10 different studies. Among the 10 genes, there are five key inflammatory genes associated with immunoglobulin E (IgE) levels, including *IL13, IL4, IL4RA, FCER1B*, and *ADRB2*.^4^, ^5^, ^6^ In our previous case–control study, we focused on nine known functional single‐nucleotide polymorphisms (SNPs) in the aforementioned five genes, and developed a four‐way genetic interaction model consisting of *IL13* rs20541, *IL4* rs2243250, *ADRB2* rs1042713, and *FCER1B* rs569108 (Table 1) for determining asthma susceptibility in Chinese Han children by using multifactor‐dimensionality reduction (MDR) approach. Each of the four SNPs had an independent effect on asthma, with rs20541 G, rs2243250 T, rs1042713 A, and rs569108 G as the risk alleles, and more risk homozygotes of the four SNPs, higher risk for asthma.^7^ Then we applied the four‐gene model among Chinese Han wheezing children and found that the high‐risk genotypes were associated with a positive Asthma Predictive Index and atopy.^8^ In a word, the four‐locus gene model has been confirmed associated with asthma and atopy in Chinese Han children.^7^, ^8^ However, whether the gene model is associated with allergies in early life has yet to be determined.

**Table 1:** Genes and SNPs constituting the four‐locus genetic model

Gene	SNP	rs Number	Chromosome position	Location
*IL13*	R110Q	rs20541	5:132660272	Exon 4
*IL4*	−590C>T	rs2243250	5:132673462	Promoter
*ADRB2*	R16G	rs1042713	5:148826877	Exon1
*FCER1B*	E237G	rs569108	11:60095631	Exon7

Abbreviations: SNP, single‐nucleotide polymorphism; rs, reference SNP.

In this study, we aimed to apply the four‐way gene model in a birth cohort with a 2‐year follow‐up to investigate associations of the model with the development of allergic diseases in Chinese Han toddlers.

## 2 METHODS

### 2.1 Study design and subjects

From 2012 to 2015, a birth cohort study was carried out at two large tertiary hospitals in Shanghai, Xinhua Hospital, and the International Peace Maternity & Child Health Hospital. One thousand and fifty‐six singleton pregnant women who planned to live in Shanghai for more than 2 years were recruited between June 2012 and February 2013. Trained research nurses conducted face‐to‐face interviews to collect information on parental atopy, family income, and parental education levels. At birth, newborns’ birth information on gender, gestational age, birth weight, delivery mode, and parity from hospital medical records along with cord blood samples were collected by trained nurses. The children were then followed up until they were 2 years old by questionnaire survey, with their epidemiologic information and medical history of allergic diseases collected. Five hundred and ninety‐seven Chinese Han children completing the 2‐year follow‐up between June 2014 and April 2015 were included in this study. Written informed consent was obtained from the participants. The study was approved by the Ethics Committee of Xinhua Hospital and the International Peace Maternity & Child Health Hospital (approval number: XHEC‐C‐2012‐003) and conducted according to the principles in the Declaration of Helsinki.

### 2.2 Two‐year follow‐up survey

Each child was followed up three times totally, at 6 months, 1 year, and 2 years of age. At 6‐month follow‐up, an internet‐based questionnaire survey was conducted to collect children's medical history of allergic diseases along with information on breastfeeding, antibiotic use, and environmental exposures, including home pet sitting, secondhand smoke exposure, and home decoration. At 1‐ and 2‐year follow‐up visits, face‐to‐face questionnaire interviews were conducted to collect similar information as that at 6‐month follow‐up.

At the three follow‐up surveys, children's caregivers were asked whether their children had been diagnosed with eczema, food allergy, wheezing, or allergic rhinitis by doctors in the past 6 months (at the 6‐month and 1‐year follow‐ups) or 1 year (at the 2‐year follow‐up). If the answer was yes, further information on eczema, wheezing or allergic rhinitis was collected by the standardized International Study of Asthma and Allergies in Childhood (ISAAC) questionnaire, which had been validated and adapted in China.^9^, ^10^ Information on food allergy was collected using a specific questionnaire, which had been developed from a previous study in China and validated by expert reviews.^11^

### 2.3 Assessment of allergic diseases

Allergic diseases assessment was made by specialists based on the questionnaires. Two dermatologists diagnosed eczema independently for all the participants, with disagreements resolved by consensus. The diagnostic criteria for eczema were: itchy, scaly rash on the flexural sites (the folds of the elbows, behind the knees or in front of the ankles), face, or around the neck or ears, and itchy rash coming and going for at least 6 months, based on the U.K. working party's diagnostic criteria.^12^

Two pediatricians specialized in respiratory medicine and allergy made diagnoses of wheezing, allergic rhinitis, and food allergy independently for all the children, and disagreements were also resolved by consensus. Wheezing was defined if children breathed with whistling sounds in the chest once or more. According to the guideline of the American Academy of Otolaryngology—Head and Neck Surgery, clinical diagnosis of allergic rhinitis was made if children had nasal congestion, runny nose, itchy nose, or sneezing when he/she did not have a cold or the flu, and it was accompanied by itchy watery eyes.^13^ As defined by the U.S. Guidelines on food allergy, if children presented with allergic symptoms after ingesting specific food, they were recorded as a parental‐reported food allergy.^14^

### 2.4 Genotyping

Genomic DNA was isolated from cord blood samples using the QIAamp DNA Blood Mini Kit (Qiagen). SNP genotyping was carried out with matrix‐assisted laser desorption/ionization‐time of flight mass spectrometry (MALDI‐TOF MS)^15^ on the MassARRAY iPLEX platform (Sequenom). The call rate for each SNP was higher than 97%. Five percent of the total samples were blindly retested and the replicate genotype concordance rate exceeded 98%.

### 2.5 Genetic risk grouping

We have found that *IL13* rs20541, *IL4* rs2243250, *ADRB2* rs1042713, and *FCER1B* rs569108 are jointly associated with asthma and atopy in Chinese Han children, and each of the SNPs has a single effect on the diseases, with rs20541 GG, rs2243250 TT, rs1042713 AA, and rs569108 GG as the risk genotypes, respectively. More risk homozygotes of the four SNPs, higher risk for asthma and atopy.^7^ As described in our previous study, the genotype combinations with no risk homozygotes (i.e., rs20541 AA or GA, rs2243250 CC or TC, rs1042713 GG or AG, and rs569108 AA or AG) were considered as the control, and those genotype combinations with a significantly higher risk for asthma and atopy (*p* < .05 and odds ratio [OR] > 1) compared with the control were categorized as high risk, otherwise as low risk.^8^ Genetic risk grouping was shown in Table 2.

**Table 2:** Genetic risk grouping based on the genotypes of the four SNPs

Group	Genotype			
	*IL13* rs20541	*IL4* rs2243250	*ADRB2* rs1042713	*FCER1B* rs569108
High risk	AA or GA	TT	GG or AG	GG
	AA or GA	TT	AA	AA or AG
	GG	CC or TC	AA	AA or AG
	GG	TT	GG or AG	AA or AG
	AA or GA	TT	AA	GG
	GG	CC or TC	AA	GG
	GG	TT	GG or AG	GG
	GG	TT	AA	AA or AG
	GG	TT	AA	GG
Low risk	AA or GA	CC or TC	GG or AG	AA or AG
	AA or GA	CC or TC	GG or AG	GG
	AA or GA	CC or TC	AA	AA or AG
	AA or GA	TT	GG or AG	AA or AG
	GG	CC or TC	GG or AG	AA or AG
	AA or GA	CC or TC	AA	GG
	GG	CC or TC	GG or AG	GG

Abbreviations: SNP, single‐nucleotide polymorphism; rs, reference SNP.

### 2.6 Statistical analysis

Each SNP was examined for Hardy–Weinberg equilibrium in the subjects by the *χ* ^2^ test. All the participants were divided into high‐ or low‐risk groups based on the genotypes of the four SNPs. The subject characteristics between the two different genetic risk groups were compared using Pearson's *χ* ^2^ or Fisher's exact test, as appropriate. Associations between genetic risk and allergic diseases were assessed by *χ* ^2^ tests, and *p* value, relative risk (RR), and 95% confidence interval (95% CI) were calculated. A two‐tailed *p* < .05 was considered statistically significant. The statistical analyses were performed using SPSS version 17.0 (IBM Corp.).

## 3 RESULTS

### 3.1 Comparison of the subject characteristics between the high and low genetic risk groups

All the SNPs met Hardy–Weinberg equilibrium criteria (*p* > .10). Among the 597 study subjects, 284 were at high risk for asthma and atopy and the others were at low risk according to the genetic risk grouping method. The subject characteristics were presented in Table 3. There was a significant difference in delivery mode between the two groups (*p* = .025). More children in the high‐risk group were born by cesarean section than those in the low‐risk group (79.2% and 71.2%, respectively). However, no significant differences were found between the two groups in gender, gestational age, birth weight, parity, breastfeeding, antibiotic use, home pet sitting, in‐home secondhand smoke exposure, home decoration, parental atopy, parental education, and family income (*p* > .05).

**Table 3:** Epidemiologic characteristics of the study subjects by genetic risk status

Phenotypes	High risk, *n* (%)	Low risk, *n* (%)	*p* value^a^
Gender			
Boy	150 (53.0)	157 (50.2)	.488
Girl	133 (47.0)	156 (49.8)	
Gestational age (wk)			
<37	10 (3.5)	11 (3.5)	.992
37–39	200 (70.4)	219 (70.0)	
≥40	74 (26.1)	83 (26.5)	
Birth weight (g)			
<2500	9 (3.2)	7 (2.2)	.480
2500–4000	247 (87.0)	282 (90.1)	
≥4000	28 (9.9)	24 (7.7)	
Delivery mode			
Vaginal	59 (20.8)	90 (28.8)	.025
Cesarean section	225 (79.2)	223 (71.2)	
Parity			
None	260 (91.5)	288 (92.0)	.837
≥1	24 (8.25)	25 (8.0)	
Breastfeeding			
No	48 (16.9)	59 (19.2)	.476
Yes	236 (83.1)	249 (80.8)	
Antibiotic use			
No	158 (55.6)	169 (54.5)	.784
Yes	126 (44.4)	141 (45.5)	
Home pet sitting^b^			
No	241 (84.9)	248 (80.0)	.121
Yes	43 (15.1)	62 (20.0)	
In‐home secondhand smoke exposure			
No	131 (46.1)	149 (48.2)	.610
Yes	153 (53.9)	160 (51.8)	
Home decoration			
No	263 (92.6)	283 (91.6)	.646
Yes	21 (7.4)	26 (8.4)	
Parental atopy^c^			
No	226 (80.4)	240 (77.7)	.412
Yes	55 (19.6)	69 (22.3)	
Maternal education			
Middle school or lower	10 (3.5)	8 (2.6)	.726
High school	32 (11.3)	39 (12.5)	
College or higher	242 (85.2)	265 (84.9)	
Paternal education			
Middle school or lower	4 (1.4)	5 (1.6)	
High school	34 (12.1)	34 (11.0)	.923
College or higher	244 (86.5)	271 (87.4)	
Family income (CNY)			
<100 K	94 (33.2)	79 (25.4)	.112
≥100 K	147 (51.9)	180 (57.9)	
Unknown	42 (14.8)	52 (16.7)	

^a^ *p* Values for χ^2^ or Fisher's exact test.

^b^ Keeping cats or dogs at home.

^c^ Parental atopy was referred to those parents who had asthma, allergic rhinitis, or atopic dermatitis along with detectable specific immunoglobulin E.

### 3.2 Associations between genetic risk and allergic diseases

As shown in Table 4, children at high genetic risk were more likely to develop eczema over 2 years of follow‐up compared with those at low risk (RR = 1.30, *p* = .044). No significant association was found between genetic risk and food allergy, wheezing, and allergic rhinitis (*p* > .05).

**Table 4:** Associations between genetic risk and allergic diseases

Group	No.	Eczema *n* (%)	Food allergy *n* (%)	Wheezing *n* (%)	Allergic rhinitis *n* (%)
High risk	284	91 (32.0%)	36 (12.7%)	18 (6.3%)	11 (3.9%)
Low risk	313	77 (24.6%)	36 (11.5%)	19 (6.1%)	12 (3.8%)
*p* value^a^		.044	.660	.892	.980
RR (95%CI)		1.30 (1.01‐1.69)	1.10 (0.72‐1.70)	1.04 (0.56‐1.95)	1.01 (0.45‐2.25)

Abbreviations: CI, confidence interval; RR, relative risk.

^a^ *p* Values for *χ* ^2^ tests.

### 3.3 Associations between genetic risk and allergic diseases after stratification for delivery mode

Table 5 shows that after stratification for delivery mode, children in the high‐risk group were still more likely to develop eczema than those in the low‐risk group (RR = 1.46, *p* = .040). And the association was even stronger than that without stratification. Still no significant associations were found between genetic risk and food allergy, wheezing and allergic rhinitis (*p* > .05).

**Table 5:** Associations between genetic risk and allergic diseases, stratified by delivery mode

Group	No.	Eczema *n* (%)	Food allergy *n* (%)	Wheezing *n* (%)	Allergic rhinitis *n* (%)
Vaginal					
High risk	59	24 (40.7%)	8 (13.6%)	4 (6.8%)	1 (1.7%)
Low risk	90	20 (22.2%)	7 (7.8%)	3 (3.3%)	4 (4.4%)
Cesarean section					
High risk	225	67 (29.8%)	28 (12.4%)	14 (6. 2%)	10 (4.4%)
Low risk	223	57 (25.6%)	29 (13.0%)	16 (7.2%)	8 (3.6%)
*P* value*		.040	.719	.956	.993
RR (95% CI)		1.46 (1.02–2.08)	1.10 (0.67–1.79)	1.02 (0.53–1.98)	1.00 (0.43–2.32)

Abbreviations: CI, confidence interval; RR, relative risk.

* *p* Values for *χ* ^2^ tests.

## 4 DISCUSSION

In our previous study, we developed a four‐way gene model including *IL13* rs20541, *IL4* rs2243250, *ADRB2* rs1042713, and *FCER1B* rs569108 for determining asthma susceptibility in Chinese Han children.^7^ Then we applied this gene model in Chinese wheezing children and found that the high‐risk genotypes were associated with a positive Asthma Predictive Index and atopy.^8^ In this study, we applied the model in a birth cohort and found that toddlers at high genetic risk were more likely to develop eczema over 2 years of follow‐up compared with those at low risk. These are a series of studies on the four‐locus gene model and the present study is the first to report the association of the gene model with the development of allergic diseases.

In this study, a significant difference in delivery mode was found between the high and low genetic risk groups, while no differences were found in other subject characteristics between the two groups. After stratification for delivery mode, the positive association between the four‐locus gene–gene interaction model with the development of eczema was a little stronger than that without stratification. In this birth cohort study, 28.14% (168/597) of the subjects developed eczema while 39.03% (233/597) developed allergies (i.e., eczema, food allergy, wheezing, or allergic rhinitis) over 2 years of follow‐up. These prevalence data were similar with those reported in other studies.^16^, ^17^

Allergic diseases have been attributed to IgE‐mediated inflammatory reactions.^18^ *IL13*, *IL4*, *ADRB2*, and *FCER1B*, which constitute the gene model, are all involved in IgE‐mediated allergic responses. Cytokines IL‐13 (IL‐13) and IL4 bind to the shared heterodimeric receptor on B cells and switch Ig production from IgM to IgE via a common signaling pathway.^19^ Allergens can stimulate beta2‐adrenergic receptors (ADRB2) on B cells and increase IgE production by a unique signaling pathway, independently of class switch recombination.^20^, ^21^ Secreted IgE from B cells binds to high‐affinity IgE receptor (FCER1) on the effector cells of allergic reactions. FCER1B, a subunit of FCER1, functions as an amplifier of FCER1 expression and signaling, which upregulates IgE‐mediated allergic responses.^22^ Each of the four SNPs in our gene model (i.e., *IL13* rs20541, *IL4* rs2243250, *ADRB2* rs1042713, and *FCER1B* rs569108) has been reported associated with increased IgE levels.^4^, ^23^, ^24^ We suppose that the four SNPs interact with each other via IgE‐mediated inflammatory pathway in the pathogenesis of allergic eczema. Future studies on the biological interactions between the SNPs are needed.

In the present study, no associations were found between the gene model and food allergy/wheezing/allergic rhinitis, which may be partly attributed to the short‐time follow‐up of the cohort and allergic diseases assessments based on parental‐reported symptoms. Hence, long‐term follow‐up and disease assessment by symptoms plus objective measurements (i.e., skin prick test or serum allergen‐specific IgE measurement) are necessary in the future.

Additionally, there are some other limitations in this study. First, only four genes (i.e., *IL13, IL4, ADRB2*, and *FCER1B*) were chosen as candidate genes. However, the four genes are susceptible genes of asthma and atopy replicated in >10 different studies,^3^ and our previous studies have found gene–gene interactions on asthma and atopy between these genes in Chinese Han children.^7^, ^8^ Second, genetic risk was classified based on the number of risk allele homozygotes, which did not fully consider the single effect of each risk allele on the disease and may weaken its role. The genetic risk score (GRS), an emerging method that integrates weak effects of each risk allele and enables valid causal estimates with large numbers of genetic variants,^25^ has been widely used in genetic studies of complex diseases.^26^, ^27^, ^28^ In our further research, more genes, and SNPs associated with allergy, as well as GRS, should be included. Third, 597 children completed 2‐year follow‐up and allergic diseases assessments were based on parental‐reported symptoms in this study. Larger samples, long‐time follow‐up, symptoms plus objective measurements, and replication of findings in other cohorts are needed in future studies.

In summary, the data presented in this study suggest that the four‐way gene model involving *IL13* rs20541, *IL4* rs2243250, *ADRB2* rs1042713, and *FCER1B* rs569108 was significantly associated with the development of eczema in Chinese Han toddlers. The four SNPs may interact with each other via IgE‐mediated inflammatory pathways in the pathogenesis of allergic eczema. Long‐term follow‐up along with functional and replication studies on the gene model are still needed in future.

## CONFLICT OF INTERESTS

The authors declare that there are no conflict of interests.

## Data Availability

The data that support the findings of this study are available from the corresponding author upon reasonable request.

## References

[1] Johansson H , Mersha TB , Brandt EB , Khurana Hershey GK . Interactions between environmental pollutants and genetic susceptibility in asthma risk. Curr Opin Immunol. 2019;60:156‐162. 31470287 10.1016/j.coi.2019.07.010 PMC6800636

[2] Hussein YM , Shalaby SM , Zidan HE , Sabbah NA , Karam NA , Alzahrani SS . CD14 tobacco gene‐environment interaction in atopic children. Cell Immunol. 2013;285:31‐37. 24044964 10.1016/j.cellimm.2013.08.001

[3] Ober C , Hoffjan S . Asthma genetics 2006: the long and winding road to gene discovery. Genes Immun. 2006;7:95‐100. 16395390 10.1038/sj.gene.6364284

[4] Liu X , Beaty TH , Deindl P , et al. Associations between total serum IgE levels and the 6 potentially functional variants within the genes IL4, IL13, and IL4RA in German children: the German Multicenter Atopy Study. J Allergy Clin Immunol. 2003;112:382‐388. 12897746 10.1067/mai.2003.1635

[5] Hizawa N , Yamaguchi E , Jinushi E , Kawakami Y . A common FCER1B gene promoter polymorphism influences total serum IgE levels in a Japanese population. Am J Respir Crit Care Med. 2000;161:906‐909. 10712341 10.1164/ajrccm.161.3.9903128

[6] Woszczek G , Borowiec M , Ptasinska A , Kosinski S , Pawliczak R , Kowalski ML . Beta2‐ADR haplotypes/polymorphisms associate with bronchodilator response and total IgE in grass allergy. Allergy. 2005;60:1412‐1417. 16197474 10.1111/j.1398-9995.2005.00869.x

[7] Hua L , Zuo XB , Bao YX , et al. Four‐locus gene interaction between IL13, IL4, FCER1B, and ADRB2 for asthma in Chinese Han children. Pediatr Pulmonol. 2016;51:364‐371. 26613553 10.1002/ppul.23322

[8] Bai S , Hua L , Wang X , Liu Q , Bao Y . Association of a 4‐locus gene model including IL13, IL4, FCER1B, and ADRB2 with the asthma predictive index and atopy in Chinese Han children. J Investig Allergol Clin Immunol. 2018;28:407‐413. 10.18176/jiaci.0272 29761786

[9] Worldwide variation in prevalence of symptoms of asthma, allergic rhinoconjunctivitis, and atopic eczema: ISAAC . The International Study of Asthma and Allergies in Childhood (ISAAC) Steering Committee. Lancet. 1998;351:1225‐1232. 9643741

[10] Chen YZ , Zhao TB , Ding Y , et al. A questionnaire based survey on prevalences of asthma, allergic rhinitis and eczema in five Chinese cities (ISAAC study). Chin J Pediatr. 1998;36:352‐355.

[11] Chen J , Liao Y , Zhang HZ , Zhao H , Chen J , Li HQ . Prevalence of food allergy in children under 2 years of age in three cities in China. Chin J Pediatr. 2012;50:5‐9. 22456067

[12] Williams HC , Jburney PG , Hay RJ , et al. The U.K. Working Party's diagnostic criteria for atopic dermatitis. I. Derivation of aminimum set of discriminators for atopic dermatitis. Br J Dermatol. 1994;131:383‐396. 7918015 10.1111/j.1365-2133.1994.tb08530.x

[13] Seidman MD , Gurgel RK , Lin SY , et al. Clinical practice guideline: allergic rhinitis. Otolaryngol Head Neck Surg. 2015;152:S1‐S43. 10.1177/0194599814561600 25644617

[14] Boyce JA , Assa'ad A , Burks AW , et al. Guidelines for the diagnosis and management of food allergy in the United States: report of the NIAID‐sponsored expert panel. J Allergy Clin Immunol. 2010;126:S1‐58. 21134576 10.1016/j.jaci.2010.10.007 PMC4241964

[15] Sharma V , Michel S , Gaertner V , et al. A role of FCER1A and FCER2 polymorphisms in IgE regulation. Allergy. 2014;69:231‐236. 24354852 10.1111/all.12336

[16] Cai J , Liu W , Hu Y , Zou Z , Shen L , Huang C . Associations between home dampness‐related exposures and childhood eczema among 13,335 preschool children in Shanghai, China: a cross‐sectional study. Environ Res. 2016;146:18‐26. 26708528 10.1016/j.envres.2015.12.009

[17] Haahtela T , von Hertzen L , Mäkelä M , Hannuksela M . Finnish allergy programme 2008–2018—time to act and change the course. Allergy. 2008;63:634‐645. 18445181 10.1111/j.1398-9995.2008.01712.x

[18] Burrows B , Martinez FD , Halonen M , Barbee RA , Cline MG . Association of asthma with serum IgE levels and skin‐test reactivity to allergens. N Engl J Med. 1989;320:271‐277. 2911321 10.1056/NEJM198902023200502

[19] Kelly‐Welch AE , Hanson EM , Boothby MR , Keegan AD . Interleukin‐4 and interleukin‐13 signaling connections maps. Science. 2003;300:1527‐1528. 12791978 10.1126/science.1085458

[20] Pongratz G , McAlees JW , Conrad DH , Erbe RS , Haas KM , Sanders VM . The level of IgE produced by a B cell is regulated by norepinephrine in a p38 MAPK‐ and CD23‐dependent manner. J Immunol. 2006;177:2926‐2938. 16920928 10.4049/jimmunol.177.5.2926

[21] Padro CJ , Shawler TM , Gormley MG , Sanders VM . Adrenergic regulation of IgE involves modulation of CD23 and ADAM10 expression on exosomes. J Immunol. 2013;191:5383‐5397. 24140643 10.4049/jimmunol.1301019 PMC3842235

[22] Kraft S , Rana S , Jouvin MH , Kinet JP . The role of the FcepsilonRI beta‐chain in allergic diseases. Int Arch Allergy Immunol. 2004;135:62‐72. 15316148 10.1159/000080231

[23] de Guia RM , Ramos JD . The ‐590C/TIL4 single‐nucleotide polymorphism as a genetic factor of atopic allergy. Int J Mol Epidemiol Genet. 2010;1:67‐73. 21537454 PMC3076745

[24] de Guia RM , Echavez MDJ , Gaw ELC , et al. Multifactor‐dimensionality reduction reveals interaction of important gene variants involved in allergy. Int J Immunogenet. 2015;42:182‐189. 25876437 10.1111/iji.12200

[25] Burgess S , Thompson SG . Use of allele scores as instrumental variables for Mendelian randomization. Int J Epidemiol. 2013;42:1134‐1144. 24062299 10.1093/ije/dyt093 PMC3780999

[26] Kawai VK , Levinson RT , Adefurin A , et al. A genetic risk score that includes common type 2 diabetes risk variants is associated with gestational diabetes. Clin Endocrinol (Oxf). 2017;87:149‐155. 28429832 10.1111/cen.13356 PMC5533106

[27] Knowles JW , Zarafshar S , Pavlovic A , et al. Impact of a genetic risk score for coronary artery disease on reducing cardiovascular risk: a pilot randomized controlled study. Front Cardiovasc Med. 2017;4:53. 28856136 10.3389/fcvm.2017.00053 PMC5558259

[28] Arabkhazaeli A , Ahmadizar F , Leusink M , et al. The association between a genetic risk score for allergy and the risk of developing allergies in childhood‐Results of the WHISTLER cohort. Pediatr Allergy Immunol. 2018;29:72‐77. 29047167 10.1111/pai.12824

